# Adaptive Habitat Selection Strategies of Human‐Breeding Red‐Crowned Cranes During the Early Stage of Rewilding

**DOI:** 10.1002/ece3.73995

**Published:** 2026-07-19

**Authors:** Yitong Tang, Hanfeng Xu, Xuenan Li, Yilin Li, Zhidong Gao, Kai Sun, Mingye Zhang, Yuandong Hu, Yingji Pan

**Affiliations:** ^1^ College of Landscape Architecture Northeast Forestry University Harbin China; ^2^ Key Laboratory of Marsh Wetland Ecosystem Conservation and Restoration, National Forestry and Grassland Administration, Northeast Institute of Geography and Agroecology Chinese Academy of Sciences Changchun China; ^3^ Key Laboratory of Wetland Ecology & National Field Observation and Research Station (Heilongjiang) for Xingkai Lake Wetland Ecosystem, Northeast Institute of Geography and Agroecology Chinese Academy of Sciences Changchun China; ^4^ Administration of Yancheng Rare Birds National Nature Reserve Yancheng China

**Keywords:** bird activities, ecological strategies, habitat preference, red‐crowned crane, spatiotemporal dynamics, wild‐release strategies

## Abstract

Wild‐release is an important approach for the recovery of rare and endangered bird populations. However, individuals released into the wild often face challenges at the early stage, including limited wild experience, unstable resource availability, and a changed habitat preference. At present, systematic quantitative analyses of the spatiotemporal dynamics of ecological adaptation in wild‐released rare and endangered birds are still limited. To fill this gap, this study focuses on six wild‐released red‐crowned cranes ( *Grus japonensis* ) in Jiangsu Yancheng National Nature Reserve for Rare Birds (YNNR). Based on 1 year of high‐temporal‐resolution GPS tracking data and the corresponding habitat information, we used spatiotemporal principal component analysis (stPCA) and Levins' niche breadth index to systematically reveal the patterns of habitat selection and habitat adaptation strategies of red‐crowned cranes during the early stage after release. The results showed that the early adaptation of wild‐released red‐crowned cranes was a dynamic process of continuous exploration, repeated adjustment, and gradual stabilization. The stPCA identified two main adaptation gradients, reflecting the change between artificial and natural habitats, and the change from reliance on artificial feeding to a resource utilization strategy enhanced by independent foraging. Individuals at different age stages exhibited significant differences in their adaptation process: immature individuals tended to adapt their wild habitats through spatial exploration and habitat use structure adjustments, showing greater overall fluctuations and an expanding niche breadth. In contrast, adult individuals were more likely to adjust their resource acquisition mode; they adapted more quickly, showed a relatively stable habitat‐use pattern, and had a smaller range of change in niche breadth. This study provides a systematic and quantitative view for evaluating the ecological adaptation process of wild‐released birds, and offers a theoretical basis and practical reference for the protection of wild‐released rare and endangered birds.

## Graphical abstract

As an endangered species and cultural symbol in China, understanding the habitat preference of rewilded red‐crowned cranes is vital for their conservation strategies. By using 36,000 GPS points, we quantified habitat preference of rewilded red‐crowned cranes in Yancheng wetlands through a quantitative spatiotemporal PCA framework. Age, season, and landscape heterogeneity drive dynamic and distinct habitat selection preference and strategies of rewilded red‐crowned cranes.

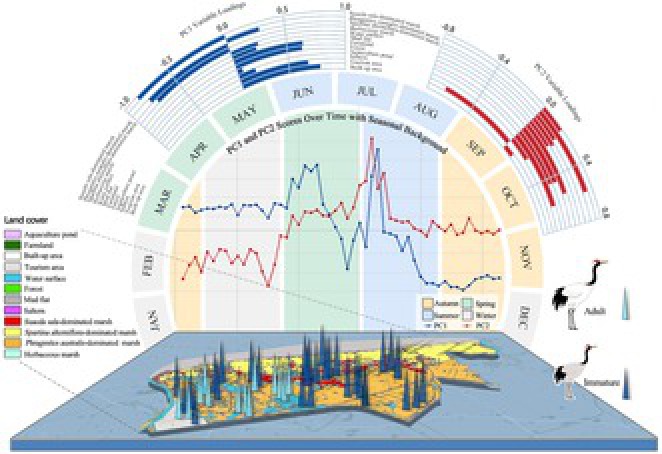

## 1 Introduction

The red‐crowned crane ( *Grus japonensis* ) is a first‐class national protected bird in China and is listed as Vulnerable (VU) by the IUCN (IUCN 2025). As a flagship species of the East Asian wetland ecosystem for its significant ecological and conservation values (Xu, Chen, et al. 2020), the survival status of red‐crowned crane serves as a vital indicator of ecosystem health (Wu et al. 2016) and plays an important role in maintaining regional and global biodiversity (Zhang et al. 2019). However, in recent years, the population of red‐crowned cranes has been threatened due to habitat degradation, human interference (Wang, Wang, et al. 2020) and climate change (Liu et al. 2020). Consequently, identifying effective scientific conservation strategies to support the recovery of the red‐crowned crane population has become a priority in current biological conservation efforts.

Wild‐release, as an important conservation strategy for endangered species (Batson et al. 2015), involves releasing captive‐bred or rehabilitated individuals back to the wild. Wild‐release has been regarded as one of the key conservation means of maintaining population size, reducing the risk of extinction, and restoring biodiversity and ecosystem functions (Kirkwood 2013). Although this measure has been widely applied in the conservation of many endangered species around the world and has made remarkable progress (Seddon et al. 2014), its successful implementation still faces many challenges. Released individuals usually encounter multiple difficulties in the early stage of release, including unfamiliar environments, limited survival skills, lack of wild experience, and failure in ecological niche occupation (Jule et al. 2008). Therefore, their survival pressure is significantly greater than that of wild‐born individuals (Cui et al. 2017). Specifically, released individuals usually exhibit greater uncertainty and variation in the process of ecological adaptation, habitat utilization, and behavioral decision‐making. Their survival strategies are not only affected by intrinsic factors such as age and physiological conditions, but also by external environmental factors including seasonal changes, human interference, and habitat heterogeneity. Therefore, continued monitoring is essential for understanding the behavior of released individuals and their interactions with habitat. Such information can improve our understanding of habitat selection strategies and help increase the effectiveness and success of wild‐release programmes (Berger‐Tal et al. 2020).

In recent years, with continued efforts to protect migratory bird flyways and core habitats for rare birds, coastal regions of eastern China, particularly the Jiangsu Yancheng National Nature Reserve for Rare Birds (YNNR), have become an important area for red‐crowned crane breeding and rewilding (Wu et al. 2024). Compared with wild populations, wild‐release red‐crowned cranes in the YNNR have smaller home ranges and are nonmigratory, requiring diverse resources within relatively fixed territories to complete their annual life cycle from breeding season to wintering season (Chen et al. 2024). So far, research on wild‐release populations of red‐crowned cranes is relatively limited, and most of the existing studies have focused on the activity range of wild‐release individuals (Cui et al. 2017), behavioral differences (Cheng et al. 2019), foraging and habitat preference between wild‐released and wild red‐crowned cranes (Wu 2017). However, there is still a lack of systematic quantitative study on how wild‐released red‐crowned cranes adapt to habitats, explore the ecological niches, and dynamically adjust their survival strategies across spatiotemporal scales in the early stage of wild‐release. Furthermore, traditional studies of released birds have mostly relied on observations or conventional radio telemetry, resulting in relatively sparse movement data (Lee et al. 2022). This makes it difficult to effectively capture the dynamic adaptation process in the early stage of release, and even more difficult to reveal the changing patterns of spatiotemporal ecological niche.

To improve the rate of successful wild‐release and provide a scientific basis for future conservation strategies, it is urgent to combine continuous GPS tracking data and quantitative ecological indicators to carry out in‐depth research on the adaptive behavior and ecological strategies of red‐crowned cranes in the early stage of release (Kays et al. 2015). This study is based on the high‐resolution GPS tracking data from six red‐crowned cranes of different ages released in the YNNR, and a total of more than 30,000 effective positioning points have been obtained (Hu et al. 2025). By interpreting their daily onsite habitat conditions, we introduce spatiotemporal Principal Component Analysis (stPCA) and niche breadth indices to systematically identify and quantify habitat preferences and niche dynamics at the early stage of wild‐release (Yanco et al. 2024). Our study aims to characterize the adaptive behavior and ecological strategies of red‐crowned cranes in the early stage of wild‐release. The findings will improve the understanding of the ecology of wild‐release red‐crowned cranes, as well as offer theoretical foundations and practical guidance for conservation, habitat optimization, and the development of age‐tailored wild‐release strategies for red‐crowned cranes.

## 2 Materials and Methods

### 2.1 Study Area

The study area is located in the YNNR in Jiangsu Province and includes the neighboring coastal wetlands, which extend from Xiangshui county in the north to Chongming district, Shanghai in the south (31°26′4.7466″ to 34°29′51.3198″ N and 119°42′0.8994″ to 121°59′10.0998″ E), covering a variety of wetland types such as natural wetlands, aquaculture ponds, salterns, farmland, and intertidal mudflats.

The YNNR is located in the transition zone from the northern subtropics to the southern warm temperate climate of East Asia. As China's largest and most ecologically diverse coastal wetland nature reserve (Xu, Liu, et al. 2019), it was designated as a Ramsar site in 2002 and inscribed on the World Heritage List in 2019. The reserve features unique flat coastlines with tidal tides and diverse tidal wetland ecosystems, with rich plant communities and animal populations; it plays a vital role as a stopover and refueling station along the East Asian—Australasian Flyway and serves as the largest wintering site for red‐crowned cranes in China (Cao et al. 2019). Each year, around three million migratory birds stop, breed, or overwinter in the region, supporting 17 threatened species listed by the IUCN (Li et al. 2024), demonstrating their exceptional global biodiversity conservation value.

### 2.2 Data Collection and Processing

#### 2.2.1 Hourly GPS Tracking Data of Red‐Crowned Cranes

Satellite tracking data were obtained from six wild‐release red‐crowned cranes of different ages that were released into the core zone of the YNNR in three batches: November 2022, February 2023, and May 2023 (Table S1). Among them, Crane No.4 and Crane No.5 are couples before wild‐release (Wu et al. 2024), while Crane No.1 and Crane No.2 were offspring of Crane No.3 (Hu et al. 2025). Before wild‐release, each red‐crowned crane was equipped with a lightweight, backpack‐style Platform Transmitter Terminal (PTT) to monitor and record bird position data at hourly intervals, with latitude, longitude, temperature, and time data. After excluding GPS points with a positioning error greater than 10 m, a total of 36,609 valid locations were retained and subsequently classified as spring (March–May), summer (June–August), autumn (September–November), and winter (December–February) (Hu et al. 2025).

#### 2.2.2 Habitat Types and Environment

##### 2.2.2.1 Data Collection

In this study, habitat identification within the study area was carried out using an integrated approach that combined Sentinel‐2 satellite image interpretation and field surveys. Sentinel‐2 images have high spatial and spectral resolution (Sánchez‐Espinosa and Schröder 2019) and show obvious advantages in identifying wetland hydrological and vegetation characteristics (Yan et al. 2022). The GPS tracking data of the red‐crowned cranes used in this study span from 2022 to 2023. In order to be consistent with the ecological processes and environmental background of this period, this study selected the Sentinel‐2 image of 2023 as the main data source for land cover classification (https://dataspace.copernicus.eu/). In addition, coastal wetland vegetation usually enters a vigorous growth period in June, and the spectral differences between different vegetation types are the most obvious (Clark 2017; Clevers and Gitelson 2013). Therefore, considering the time span of GPS tracking data, the phenological characteristics of coastal vegetation, and satellite overpass time and cloud conditions, eight Sentinel‐2 images in 2023 were finally selected to cover the entire study area. Such data selection not only helps to ensure the accuracy of land cover classification during the study period, but also improves the temporal consistency between the extracted results with the actual ecological conditions of red‐crowned cranes.

##### 2.2.2.2 Data Processing

**FIGURE 1:**
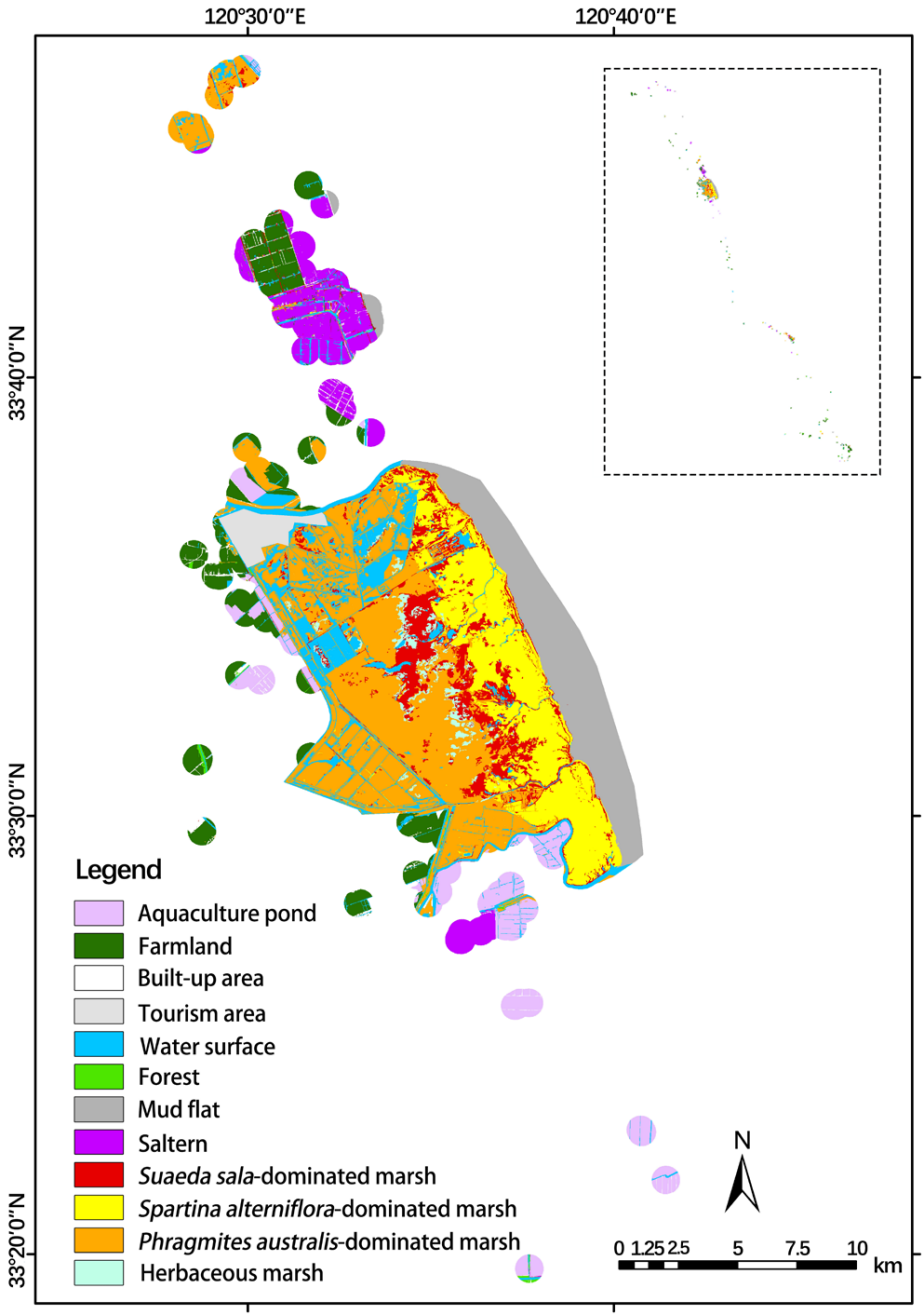
Landscape and land cover of the red‐crowned crane activity sites.

Red‐crowned cranes exhibit continuous movement in the wild, and a single GPS location is therefore difficult to use to accurately reflect their actual habitat utilization. To improve the efficiency of remote sensing interpretation, this study adopts a target‐centered local land cover classification method, that is, a buffer zone is established around each GPS location to extract habitat information related to red‐crowned crane activities. The radius of each buffer is determined by calculating the distances between consecutive hourly GPS locations. In order to capture the upper limit of the typical hourly movement distance and reduce the interference of a few outliers, this study adopts the 90th percentile (600 m) of these movement distances as the buffer radius (Johnson and Seip 2008; Tost et al. 2022). If the maximum movement distance were adopted, the spatial scale of conventional habitat utilization may be overestimated due to extreme values, because such long‐distance movements are often only occasional events and do not represent the typical behavior of cranes. In contrast, the average or median mainly reflects the central tendency of movement distance, which may underestimate the spatial range of red‐crowned cranes interacting with surrounding habitats during the active period. Thus, this study regards 600 m as the indicator value of the potential foraging and habitat utilization scale of red‐crowned cranes under this time resolution, and further extracts the land cover types in each buffer zone for subsequent analysis (Figure 1).

In terms of technical methods, this study adopts the supervised classification method for land cover interpretation. Atmospheric correction, image mosaicking, and clipping of remote sensing image were performed using the ENVI 5.6 software platform (Harris Geospatial Solutions Inc. 2020), then, complete the land cover classification with the help of eCognition Developer 9.0 (Trimble Germany GmbH 2014) and ArcGIS 10.7 (Esri 2019), and the classification results were further revised and optimized in combination with field survey data. The accuracy evaluation results based on the confusion matrix showed that the overall classification accuracy was 86.81% and the Kappa coefficient was 84.76%, indicating a high level of reliability in the classification results. The final land cover map meets the accuracy requirements of the habitat selection analysis of red‐crowned cranes, and provides a reliable data basis for subsequent research (McHugh 2012).

The final land coverage classification includes a total of 12 habitat types (Figure 1), which were broadly classified into three categories: natural wetland habitats, production landscapes, and human‐modified habitats. The specific habitat types included in each category, and their definitions and ecological significance, are presented in Table S2. It should be particularly noted that tourism area in this study mainly refers to visitor‐use areas within the core zone and their surrounding spaces. This habitat type not only represents areas with relatively concentrated human activity, but is also closely associated with supplementary feeding sites and other forms of human‐provided resources. Because habitat use in these areas is difficult to interpret independently from the effects of supplementary feeding, tourism area and its surrounding spaces were extracted separately and classified as an independent habitat category in this study, in order to improve the ecological interpretability and robustness of habitat‐use analyses.

### 2.3 Data Analysis

To systematically analyze habitat preference patterns and ecological adaptation strategies of red‐crowned cranes during the early stage of wild‐release, this study used spatiotemporal principal component analysis (stPCA) as the main analytical method (Yanco et al. 2024), with Levins' niche breadth index (Levins 1968) as a complementary metric. Unlike traditional PCA, which is usually limited to a single temporal or spatial scale, stPCA can simultaneously capture temporal dynamics and variation in habitat‐use structure. Accordingly, stPCA was used to identify the dominant ecological gradients and temporal dynamics of habitat‐use change, whereas Levins' niche breadth quantified variation in resource‐use breadth and evenness. Habitat composition within buffers around hourly GPS locations was first extracted as the proportions of 12 land‐cover types and then aggregated at the weekly scale to reduce short‐term fluctuations and spatiotemporal autocorrelation associated with high‐frequency tracking data (Yanco et al. 2024). Given the limited sample size and to reduce the influence of extreme values from particular individuals, analyzed were conducted at the age‐class level rather than separately. Based on age records at release, individuals aged 0.5–3 years were classified as the immature group, including juvenile and subadult cranes, whereas individuals aged 8–10 years were classified as the adult group. For each age class, weekly median proportions of the 12 habitat types were used to construct group‐level spatiotemporal matrices for stPCA. The same weekly group‐level habitat proportions were also used to calculate Levins' niche breadth index. Together, stPCA and Levins' niche breadth index characterized early post‐release adaptation from two complementary perspectives: changes in habitat‐use structure and changes in resource‐use breadth. Detailed procedures are provided in Table S3. The weekly principal component scores were calculated as follows:

$${\mathrm{PC}}_{k}^{\left(i\right)}=\sum_{j=1}^{p}a_{\mathrm{kj}}z_{\mathrm{ij}}$$

where $z_{\mathrm{ij}}$ is the standardized proportion of habitat type j in week i, and $a_{\mathrm{kj}}$ is the loading of variable j on the k‐th principal component.

The breadth of ecological niches is widely recognized as an important indicator of environmental adaptability and a key factor influencing species distribution and survival strategies (Di Cecco and Hurlbert 2022). Therefore, this study incorporated the Levins' niche breadth index (Levins 1968) as a complementary analysis to quantify the degree of habitat specialization or generalization of red‐crowned cranes. The index effectively measures the evenness and diversity of resource use, thereby reflecting ecological flexibility and the potential for niche expansion (Colwell and Futuyma 1971). The index is calculated as follows:

$$B=\frac{1}{\sum_{i=1}^{n}p_{i}^{2}}$$

where B denotes niche breadth, $p_{i}$ represents the proportion of crane habitat use in habitat type i (by analogy with stPCA, the mean proportions of each type of land cover were calculated weekly to derive $p_{i}$), and n is the total number of habitat types. The higher value of B indicates that the individual or population uses a broader range of habitat types more evenly, suggesting greater ecological flexibility and a wider niche. On the contrary, a lower B value suggests a more concentrated use of resources and a narrower breadth of niches.

## 3 Results

### 3.1 Age‐Dependent Differences in Dominant stPCA Adaptation Gradients and Adaptation Dynamics

**FIGURE 2:**
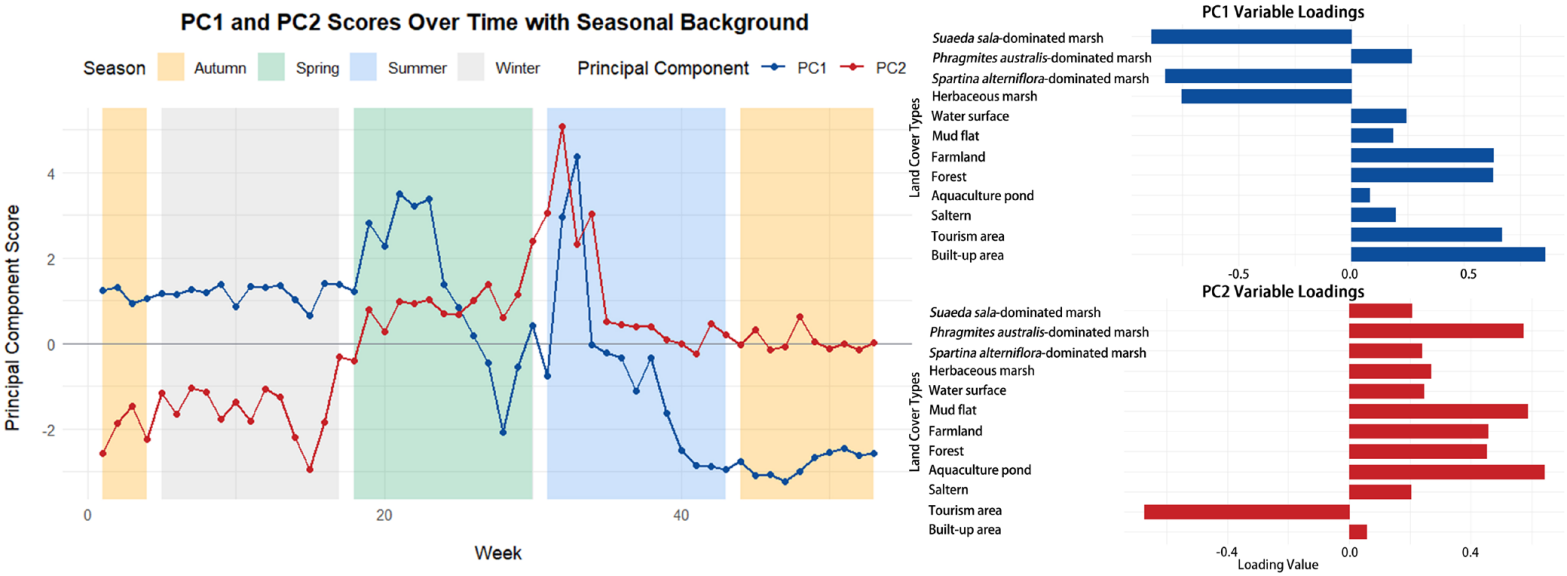
Principal component scores and load scores of the immature group.

**FIGURE 3:**
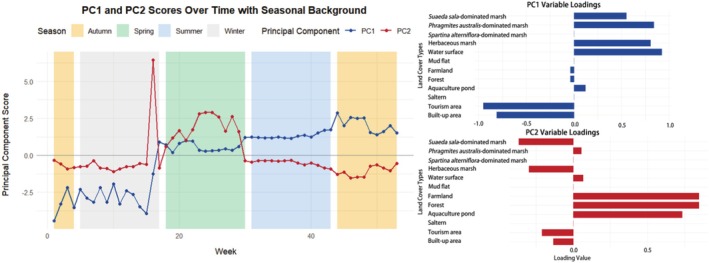
Principal component scores and load scores of the adult group.

The loading structures differed between the immature and adult groups (Figures 2 and 3). In the immature group, PC1 was positively associated with built‐up area, tourism area, farmland, and forest, and negatively associated with *Suaeda salsa*, *Spartina alterniflora*, and herbaceous marshes, representing an “artificial habitat–natural habitat” gradient. In contrast, PC1 of the adult group was positively associated with water surface, *Phragmites australis*, *Suaeda salsa*, and herbaceous marshes, and negatively associated with tourism and built‐up areas, representing a “supplementary feeding space–diversified foraging habitat” gradient. For PC2, the immature group showed negative associations with tourism area and positive associations with aquaculture ponds, mudflats, and *Phragmites australis* marsh, representing a “supplementary feeding space–diversified foraging habitat” gradient and reflecting a transition from supplementary feeding to autonomous foraging across diverse habitats. Conversely, PC2 of the adult group was positively associated with farmland, forest, and aquaculture ponds and negatively associated with *Suaeda salsa* and herbaceous marshes, representing an “artificial habitat–natural habitat” gradient. Overall, the dominant gradients were reversed between age groups: Habitat‐use shifts between artificial and natural habitats were captured primarily by PC1 in the immature group but by PC2 in the adult group, whereas the supplementary feeding–foraging transition was represented by PC2 in the immature group and PC1 in the adult group.

Changes in principal component scores revealed contrasting adaptation dynamics between age groups (Figures 2 and 3). The immature group showed a gradual shift from artificial habitats and supplementary feeding spaces toward natural wetlands and autonomous foraging, whereas adults exhibited a more rapid transition from supplementary feeding‐related spaces to natural wetland habitats, stabilizing by early spring. Seasonal fluctuations in PC2 further suggest that adults adjusted habitat use in response to changing resource conditions while maintaining consistent use of natural wetlands. Overall, both age groups increasingly relied on natural habitats, but adaptation in immature individuals was driven primarily by habitat‐use restructuring, whereas adults were characterized by a rapid shift in resource acquisition strategy followed by earlier stabilization.

### 3.2 Age‐Dependent Differences in Niche Breadth Dynamics

**FIGURE 4:**
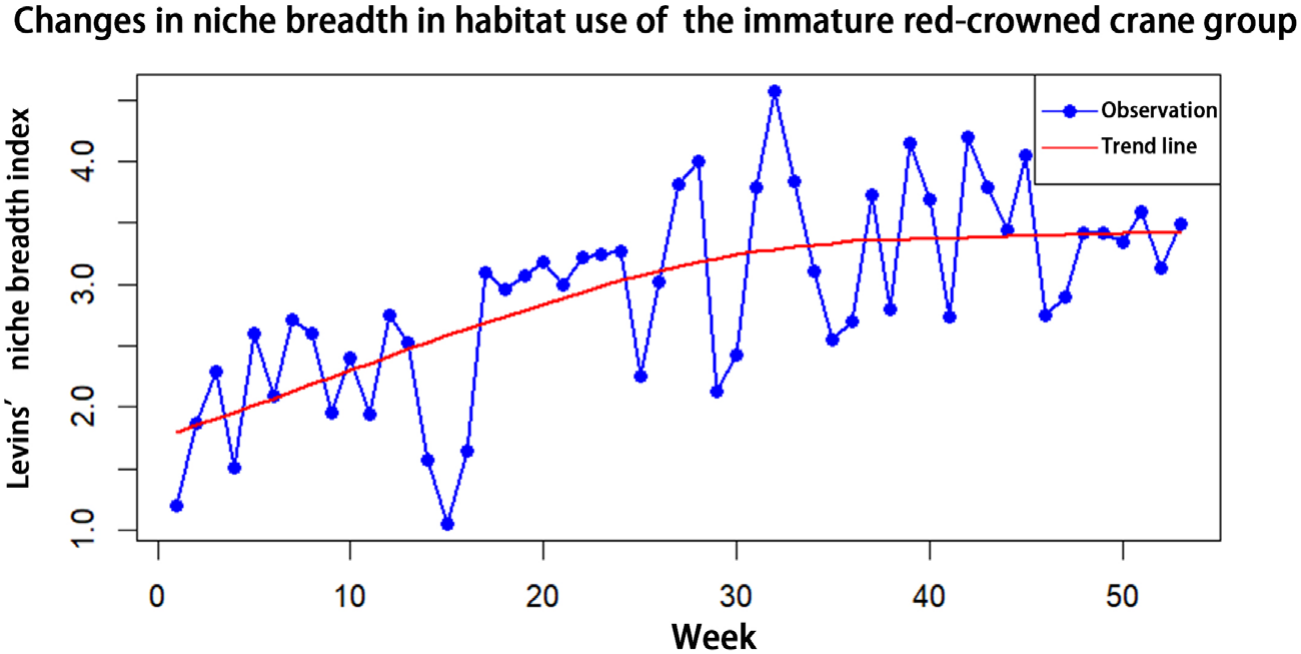
Changes in niche breadth in habitat use of the immature group.

Levins' niche breadth index showed differences between the immature and adult groups in the breadth and stabilization of resource use. In the immature group (Figure 4), niche breadth was relatively low during the early stage after release, indicating a limited range of habitat and resource use. As time after release increased, niche breadth generally increased and showed relatively clear fluctuations, suggesting that the immature group gradually expanded its use of different habitat types and resource spaces. Although niche breadth still fluctuated during the later stage, it was generally maintained at a relatively stable level.

**FIGURE 5:**
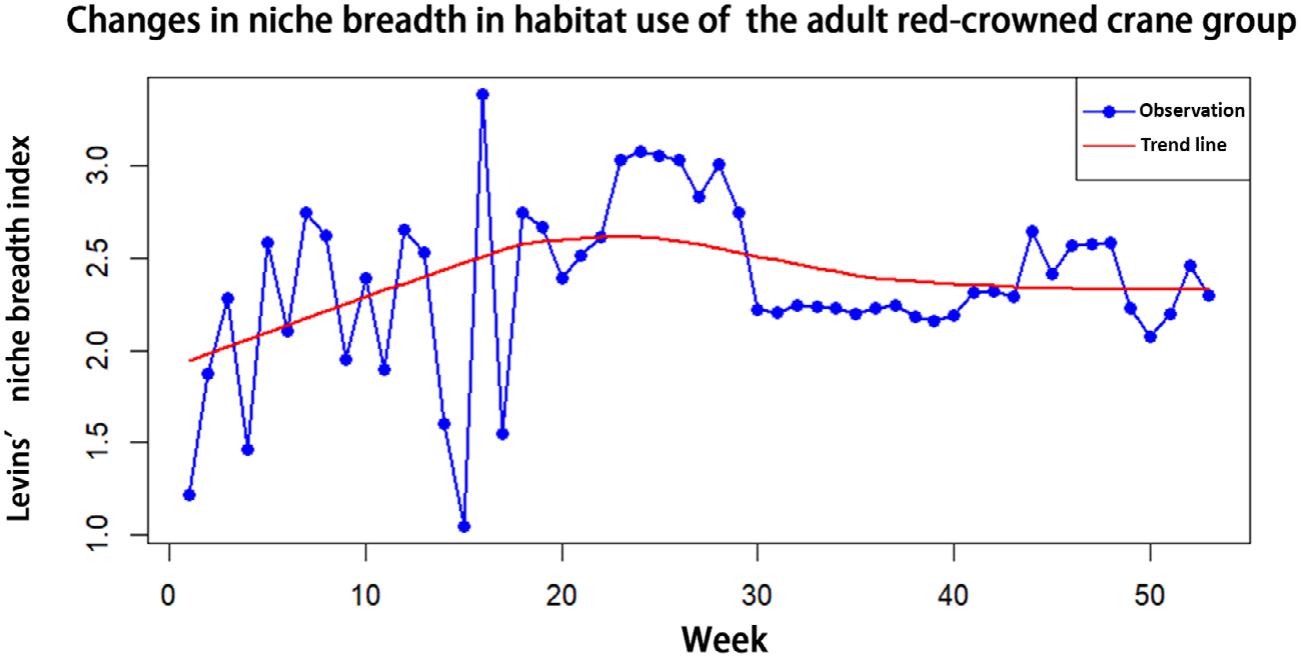
Changes in niche breadth in habitat use of the adult group.

In the adult group (Figure 5), niche breadth showed a more stable overall pattern. Niche breadth fluctuated during the early stage after release, indicating that the adult group still adjusted its use of different habitat types during the initial adaptation period. It then gradually became smoother and more stable, suggesting that a relatively stable breadth of resource use had formed. Compared with the immature group, the adult group showed a smaller overall range of change in niche breadth, indicating a faster stabilization of resource use.

## 4 Discussion

This study examined the habitat adaptation process of wild‐released red‐crowned cranes during the early stage after release. The results showed that released individuals generally shifted from artificial support spaces toward increased use of natural wetlands, but the dominant adaptation pathways differed between immature and adult cranes. Immature individuals showed stronger spatial exploration, restructuring of habitat‐use patterns, and expansion of niche breadth, whereas adult individuals shifted more rapidly in resource acquisition mode and formed a relatively stable habitat‐use pattern earlier. Habitat adaptation mainly developed along two integrated ecological gradients: the artificial habitat–natural wetland gradient and the supplementary feeding–autonomous foraging gradient. These findings suggest that early post‐release adaptation is not a direct selection of a single suitable habitat, but a gradual adjustment of resource use and spatial use under the combined effects of age‐stage differences and functional habitat combinations.

### 4.1 Age‐Dependent Differences in Dominant Post‐Release Adaptation Strategies of Red‐Crowned Cranes

Age stage may be an important factor influencing the early adaptation strategies of wild‐released red‐crowned cranes. Based on the changes in principal component scores and niche breadth, immature cranes appeared to adapt mainly by exploring different habitats, expanding the breadth of resource use, and gradually screening suitable spaces. In contrast, adult cranes tended to adjust more rapidly around key food resources and formed stable use patterns earlier. This difference may be related to age‐related differences in spatial experience, risk assessment, and resource acquisition ability.

Previous studies have shown that age can affect foraging efficiency, vigilance behavior, information use and spatial decision‐making in birds (Li et al. 2013; Wang et al. 2009; Mueller et al. 2013). Immature individuals usually lack mature foraging experience and spatial recognition ability, and are therefore more likely to accumulate environmental information through broader exploration. During the early post‐release stage, they may also undergo a prolonged wandering period to accumulate spatial experience and optimize habitat selection (Mirski et al. 2025). In contrast, adult birds are more likely to use previous experience for efficient resource selection (Franks and Thorogood 2018). At the same time, avoidance of novel stimuli may increase with age, which may also explain why younger birds often show stronger exploratory behavior in unfamiliar environments (O'Hara et al. 2017). Consistent with this age‐related pattern, immature red‐crowned cranes exhibit greater spatial exploration than adults during the early post‐release period (Cui et al. 2017).

Therefore, the stronger exploratory tendency observed in the immature group may reflect a process in which individuals established spatial knowledge, compared resource availability, and screened suitable habitats through contact with different habitats and resource spaces during the early stage after release. In comparison, because adult cranes have relatively mature foraging experience and risk recognition ability, they may be more likely to form a stable use pattern around key resources soon after entering a new environment. Thus, age differences may shape the dominant adaptation strategies of wild‐released red‐crowned cranes by influencing spatial learning, resource recognition, and the adaptation process.

### 4.2 Functional Habitat Combinations Reflected by Dominant Adaptation Gradients

The two dominant adaptation gradients indicate that habitat adaptation in released red‐crowned cranes was driven not by any single habitat type but by an integrated response to habitat combinations with similar ecological functions. The key habitats involved can be grouped into three functional categories, namely supplementary feeding spaces, production landscapes, and natural wetlands, which played complementary roles and collectively formed the spatial basis for adaptation. Among these habitats, supplementary feeding spaces represented by tourism areas primarily served as initial buffering habitats. Newly released individuals need to cope with unfamiliar environments, resource uncertainty, and behavioral adjustment pressures, and stable food resources may help reduce these short‐term risks, explaining their association with supplementary feeding spaces after release. Previous studies have similarly shown that supplementary feeding can improve survival and spatial stability during the initial post‐release period, although prolonged or intensive feeding may lead to behavioral dependence, spatial aggregation, and reduced development of natural foraging abilities (Ewen et al. 2015). Therefore, supplementary feeding spaces should be managed as transitional support habitats, with feeding intensity, location, and duration gradually adjusted to minimize long‐term dependence (Greggor et al. 2024).

Production landscapes, such as farmland and aquaculture ponds, played a supplementary resource role. These spaces are not equivalent to direct artificial support, but they may provide additional resources when natural resources are limited or when individuals are still in the exploratory stage. For example, harvested farmland may provide leftover grain resources (Lee 2018), while aquaculture ponds may provide fish, shrimp, and other aquatic animal resources (Xu, Zhang, et al. 2020; Wang, Li, et al. 2020). However, production landscapes are usually associated with higher levels of human disturbance. Their use value depends on resource availability, disturbance intensity, and spatial connectivity with natural wetlands. Therefore, management of these spaces should focus on controlling human disturbance and maintaining spatial connectivity with natural wetlands, in order to reduce the risks faced by released individuals during stage‐specific use.

Natural wetlands are the core habitats that support long‐term autonomous foraging and stable space use. Wetland combinations represented by *Phragmites australis* ‐dominated marsh, *Suaeda salsa*‐dominated marsh, and water surface usually contain a mosaic of shallow water, vegetation, and food resources (Ma et al. 1999; Xu, Zhang, et al. 2019). These habitats can provide aquatic plants, fish, snails, and other invertebrates for red‐crowned cranes (Luo et al. 2015). Compared with supplementary feeding spaces and production landscapes, natural wetlands have greater ecological stability and lower dependence on human support, and therefore provide a stronger basis for long‐term autonomous foraging and stable spatial use by released individuals (Van Schmidt et al. 2014).

Overall, these three functional habitat categories jointly form a continuous adaptive environment through which released red‐crowned cranes can shift from artificial dependence toward natural habitat use. Therefore, management should coordinate the gradual withdrawal of supplementary feeding, risk control in production landscapes, and improvement of natural wetland quality according to the roles of different functional spaces in the post‐release adaptation process.

### 4.3 Management Implications for Age‐Tailored Wild‐Release

The above results indicate that wild‐release management should not evaluate success only by short‐term survival after release (Stone et al. 2025). It should also consider whether individuals can gradually reduce their dependence on artificial support and develop relatively stable patterns of resource use and habitat selection in natural environments (Bubac et al. 2019; Drenske et al. 2023). Given the influence of age on adaptation strategies and stabilization rates, management should adopt age‐tailored measures and establish a dynamic evaluation mechanism based on continuous monitoring (Kirkwood 2013; Armstrong et al. 2017).

For immature individuals, management should focus on reducing exploration‐related risks and providing safe and continuous transitional environments for spatial learning. Strong exploratory behavior can help immature cranes accumulate environmental information, but it may also increase their exposure to human disturbance and unsuitable habitats. Therefore, during the early stage after release, management should maintain low‐disturbance conditions around the release site and retain transitional landscapes that are continuously connected with natural wetlands. Tracking data should also be used to identify abnormal dispersal, excessive dependence on artificial spaces, or prolonged use of high‐risk areas, thereby providing a basis for necessary management interventions (Urbanek et al. 2010). For adult individuals, management should focus more on maintaining the quality of key natural foraging spaces. Adult cranes are more likely to form stable spatial‐use patterns around available resources relatively quickly. Therefore, open shallow water areas, wetland vegetation mosaics, and natural food resources around the release site should be maintained as a priority to improve resource availability and accessibility of foraging spaces (Cao et al. 2015). At the same time, management should continue to assess whether adult individuals gradually reduce their dependence on artificial support spaces and form relatively fixed natural foraging and habitat‐selection patterns.

Age differences also suggest that the age composition of release groups should receive greater attention in release planning. Previous studies have shown that age can affect post‐release survival, dispersal, and settlement outcomes in birds (Li et al. 2022; Miller et al. 2024). Future release practices could further evaluate how different age combinations influence release success, spatial dispersal, and the development of natural foraging ability. For example, whether a higher proportion of adult individuals can improve group activity stability, and whether different age combinations alter post‐release dispersal and settlement processes, remain questions that require further testing (Kuwabara et al. 2024). Overall, age‐tailored management should be combined with continuous post‐release tracking, so that management measures can be adjusted according to the actual adaptation status of individuals rather than being evaluated only by fixed time points or uniform criteria.

### 4.4 Limitations

This study still has several limitations. Because wild‐release practices and long‐term monitoring of rare and endangered birds are constrained by practical conditions, the number of released individuals with continuous GPS tracking data was limited, and both sample size and monitoring duration were restricted. Nevertheless, the data used in this study were obtained from real released individuals and had strong continuity and high temporal resolution. They therefore provide useful support for identifying spatial use, habitat adaptation, and resource‐use adjustment during the early stage after release. To reduce the influence of individual variation on interpretation, this study compared patterns across age stages, seasonal changes, and habitat‐use dynamics, and remained cautious in interpreting and generalizing the results. Therefore, the conclusions of this study are more applicable to understanding and evaluating behavioral responses and habitat adaptation processes during the early post‐release stage. Future studies should continue tracking more released individuals and additional release batches, and integrate data on water‐level dynamics, food resources, human disturbance, and direct behavioral observations. Such data would help further test the mechanisms driving habitat selection and adaptation processes in released individuals.

Despite these limitations, this study suggests that age‐dependent adaptation pathways, functional habitat gradients, and the stabilization of resource use are important entry points for understanding the early adaptation of wild‐released red‐crowned cranes, and they also provide a targeted analytical perspective for subsequent wild‐release management and habitat optimization.

## 5 Conclusions

This study revealed the adaptive habitat selection strategies adopted by red‐crowned cranes during the early stage after wild‐release. The results showed that both the immature and the adult exhibited an overall trend of shifting from artificial support to increased use of natural wetlands, but the immature group was more inclined to adjust habitat‐use structure, whereas the adult group showed a stronger tendency to shift resource acquisition strategies. At the same time, the breadth of resource use in the immature group generally expanded and then became stable, whereas the adult group showed smaller fluctuations and an overall stable trend, reflecting differences in the pace of adaptation and patterns of habitat use between age stages during the early stage after wild‐release. Future research should combine long‐term behavioral monitoring with experimental validation of diversified wild‐release strategies to further deepen understanding of wild‐release pathways and improve related ecological management. This study provides quantitative tools and empirical support for understanding shifts in post‐release survival strategies, deepens understanding of the mechanisms of red‐crowned cranes adaptation during the early stage after wild‐release, and offers theoretical reference and practical guidance for the wild‐release and habitat management of endangered species.

## Author Contributions

**Yitong Tang:** conceptualization (equal), data curation (lead), formal analysis (lead), methodology (equal), software (equal), writing – original draft (lead). **Hanfeng Xu:** investigation (equal), validation (equal), writing – review and editing (equal). **Xuenan Li:** investigation (supporting), methodology (equal), software (lead), validation (equal). **Yilin Li:** investigation (equal), validation (equal). **Zhidong Gao:** data curation (equal), investigation (equal), resources (lead), validation (equal). **Kai Sun:** data curation (supporting), investigation (equal). **Mingye Zhang:** investigation (equal), methodology (equal), validation (equal). **Yuandong Hu:** supervision (equal), funding acquisition (equal), writing – review and editing (equal). **Yingji Pan:** conceptualization (lead), funding acquisition (lead), methodology (equal), project administration (lead), supervision (lead), writing – review and editing (lead).

## Funding

This work was supported by National Key Research and Development Program of China, 2023YFF1304502. The Talents Program of the Chinese Academy of Sciences, E429S101. The Innovation Team Project of Northeast Institute of Geography and Agroecology, Chinese Academy of Sciences, 2023CXTD03.

## Conflicts of Interest

The authors declare no conflicts of interest.

## Supporting information


**Data S1:** ece373995‐sup‐0001‐DataS1.R.

**Data S2:** ece373995‐sup‐0002‐DataS2.R.

**Data S3:** ece373995‐sup‐0003‐DataS3.R.

**Data S4:** ece373995‐sup‐0004‐DataS4.R.

**Table S1:** GPS tracking data of the six red‐crowned cranes released in wild in Jiangsu Yancheng National Nature Reserve for Rare Birds (YNNR), China.
**Table S2:** Definition and ecological description of habitat types in the study area.
**Table S3:** (a) Spatiotemporal matrix of weekly habitat use in the immature group of red‐crowned cranes. (b) Spatiotemporal matrix of weekly habitat use in the adult group of red‐crowned cranes.

## Data Availability

The remote sensing data used in this study are available at Copernicus Data Space Ecosystem (https://dataspace.copernicus.eu/). GPS tracking data of red‐crowned cranes can be accessed in the PANGAEA data repository (https://doi.pangaea.de/10.1594/PANGAEA.968698).
