# Supplementary material for: Adaptive Habitat Selection Strategies of Human‐Breeding Red‐Crowned Cranes During the Early Stage of Rewilding
Source: Ecol Evol. 2026 Jul 19;16(7):e73995. doi: 10.1002/ece3.73995 (PMC13381066; doi:10.1002/ece3.73995)
Supplement: Supplementary file 5 — Table S1: GPS tracking data of the six red‐crowned cranes released in wild in Jiangsu Yancheng National Nature Reserve for Rare Birds (YNNR), China. Table S2: Definition and ecological description of habitat types in the study area. Table S3: (a) Spatiotemporal matrix of weekly habitat use in the immature group of red‐crowned cranes. (b) Spatiotemporal matrix of weekly habitat use in the adult group of red‐crowned cranes. [file ECE3-16-e73995-s001.docx]

**Supplementary Material for Adaptive habitat selection strategies of human-breeding red-crowned cranes during the early stage of rewilding**

**Table S1** GPS tracking data of the six red-crowned cranes released in wild in Jiangsu Yancheng National Nature Reserve for Rare Birds (YNNR), China.

| No. | Wild-Release date | Sex | Age (a) | Age class | End date |
| --- | --- | --- | --- | --- | --- |
| 1 | 2022.11.03 | NA | 0.5 | Juvenile | 2023.11.5 |
| 2 | 2022.11.03 | NA | 0.5 | Juvenile |  |
| 3 | 2022.11.03 | Female | 8 | Adult |  |
| 4 | 2023.02.16 | Female | 10 | Adult |  |
| 5 | 2023.02.16 | Male | 10 | Adult |  |
| 6 | 2023.05.05 | Male | 3 | Subadult |  |

**Table S2** Definition and ecological description of habitat types in the study area

| **Habitat type** | **Definition** | **Ecological Significance** |
| --- | --- | --- |
| *Suaeda sala*-dominated marsh | Marsh habitat dominated by *Suaeda salsa*, typically occurring in coastal saline-alkaline wetlands. | Typical coastal salt marsh habitat, providing natural foraging and roosting space under relatively low disturbance. |
| *Phragmites australis*-dominated marsh | Marsh habitat dominated by *Phragmites australis*, usually characterized by relatively tall emergent vegetation. | Typical coastal salt marsh habitat, providing some concealment and foraging space under relatively low disturbance. |
| *Spartina alterniflora*-dominated marsh | Marsh habitat dominated by *Spartina alterniflora*, a coastal intertidal vegetation type often forming dense stands | A coastal wetland vegetation habitat; dense vegetation may reduce accessibility and foraging efficiency, giving it a mixed ecological role. |
| Herbaceous marsh | Marsh habitat dominated by herbaceous plants other than *Suaeda salsa*, *Phragmites australis*, or *Spartina alterniflora.* | Other herb-dominated marshes, providing shallow water, herb cover, and small food resources, and representing part of the diverse natural wetland habitat. |
| Water surface | Open and relatively stable water-covered habitat, including rivers, ponds, lakes, and other open water bodies. | Open water habitat, which can serve as drinking, resting, and transitional activity space, and together with surrounding shallow wetlands forms an important activity environment. |
| Mud flat | Exposed intertidal muddy or silty flat, usually lacking dense vegetation cover. | Exposed intertidal mudflat with high openness and visibility, providing natural food resources such as benthic animals and serving as an important natural foraging habitat. |
| Farmland | Land primarily used for agricultural production, including cropland under cultivation, fallow, or post-harvest conditions. | May provide residual grain, invertebrates, and temporary activity space after harvest, during flooding, or in fallow periods, but its use is strongly seasonal and easily affected by farming disturbance. |
| Forest | Land dominated by trees or woody vegetation, forming relatively closed vegetation cover compared with open wetlands. | Compared with open wetlands, forest is generally not a preferred habitat for red-crowned cranes and more often reflects a low-suitability or restrictive habitat background. |
| Aquaculture pond | Artificial pond or impoundment used for aquaculture production, such as fish or shrimp farming. | Artificial ponds used for aquaculture that may provide supplementary food resources and shallow-water activity space, but whose use is strongly influenced by management and human disturbance. |
| Saltern | Artificial wetland or pond system used for salt production through seawater evaporation and brine management. | Artificial wetland used for salt production; may provide shallow water, bare ground, and water–land transitional space under certain conditions, but resource availability depends on production cycles and management. |
| Tourism area | Area associated with tourism, visitor activities, management facilities, or supplementary feeding sites. | Represents not only areas of relatively concentrated human activity, but also spaces closely associated with supplementary feeding and other human-provided resources; in this study, it mainly reflects habitat use under artificial feeding conditions. |
| Built-up area | Land occupied by buildings, roads, infrastructure, and other highly modified human-made surfaces. | Usually represents areas with strong human activity and high land modification, generally unsuitable as core crane habitat, mainly reflecting increased disturbance and reduced habitat suitability. |

**Ecological interpretation of habitat types:** Natural wetland habitats mainly include *Suaeda salsa*-dominated marsh, *Phragmites australis*-dominated marsh, *Spartina alterniflora*-dominated marsh, herbaceous marsh, water surface, and mud flat, which generally provide the basic conditions required by red-crowned cranes for foraging, roosting, and daily activities. Production landscapes, including aquaculture pond, saltern, and farmland, may provide supplementary food resources or transitional activity space under certain conditions, but their availability is often strongly influenced by management practices and is usually associated with greater human disturbance. Human-modified habitats, including tourism area, built-up area, and forest, mainly reflect habitat contexts with stronger human influence or generally lower suitability. Overall, in interpreting the habitat-use dynamics of red-crowned cranes, these 12 habitat types were treated not only as land-cover classes, but also as habitat contexts with different ecological functions and disturbance backgrounds.

**Table S3**

(a) Spatio-temporal matrix of weekly habitat use in the immature group of red-crowned cranes

| Week | *S.salsa* marsh | *P.australis* marsh | *S.alterniflora* marsh | Water surface | Aquaculture pond | Herbaceous marsh | Farmland | Saltern | Built-up area | Forest | Mud flat | Tourism area |
| --- | --- | --- | --- | --- | --- | --- | --- | --- | --- | --- | --- | --- |
| 1 | 0.23 | 3.41 | 0.00 | 4.33 | 0.46 | 0.00 | 0.15 | 0.00 | 0.07 | 0.00 | 0.00 | 91.36 |
| 2 | 0.80 | 15.04 | 0.00 | 13.32 | 0.00 | 0.00 | 0.22 | 0.00 | 0.08 | 0.00 | 0.00 | 70.54 |
| 3 | 1.78 | 25.50 | 0.00 | 13.26 | 0.00 | 0.00 | 0.00 | 0.00 | 0.05 | 0.00 | 0.00 | 59.40 |
| 4 | 0.97 | 11.45 | 0.00 | 7.92 | 0.00 | 0.00 | 0.01 | 0.00 | 0.06 | 0.00 | 0.00 | 79.60 |
| 5 | 1.22 | 29.51 | 0.00 | 17.35 | 0.00 | 0.00 | 0.00 | 0.00 | 0.07 | 0.00 | 0.00 | 51.85 |
| 6 | 0.78 | 21.59 | 0.00 | 13.11 | 0.00 | 0.00 | 0.00 | 0.00 | 0.07 | 0.00 | 0.00 | 64.45 |
| 7 | 1.36 | 31.42 | 0.00 | 18.31 | 0.00 | 0.00 | 0.00 | 0.00 | 0.09 | 0.00 | 0.00 | 48.82 |
| 8 | 1.43 | 29.99 | 0.00 | 17.30 | 0.00 | 0.00 | 0.00 | 0.00 | 0.08 | 0.00 | 0.00 | 51.20 |
| 9 | 1.08 | 18.86 | 0.00 | 11.99 | 0.01 | 0.00 | 0.01 | 0.00 | 0.10 | 0.00 | 0.00 | 67.96 |
| 10 | 2.59 | 27.40 | 0.00 | 12.87 | 0.00 | 0.00 | 0.01 | 0.00 | 0.04 | 0.00 | 0.00 | 57.08 |
| 11 | 1.07 | 17.65 | 0.00 | 12.60 | 0.00 | 0.00 | 0.00 | 0.00 | 0.09 | 0.00 | 0.00 | 68.59 |
| 12 | 1.27 | 28.78 | 0.00 | 21.52 | 0.00 | 0.00 | 0.00 | 0.00 | 0.09 | 0.00 | 0.00 | 48.34 |
| 13 | 1.03 | 26.06 | 0.00 | 19.06 | 0.00 | 0.00 | 0.00 | 0.00 | 0.10 | 0.00 | 0.00 | 53.75 |
| 14 | 0.40 | 11.57 | 0.00 | 9.61 | 0.00 | 0.00 | 0.00 | 0.00 | 0.05 | 0.00 | 0.00 | 78.36 |
| 15 | 0.04 | 0.36 | 0.00 | 2.20 | 0.00 | 0.00 | 0.00 | 0.00 | 0.00 | 0.00 | 0.00 | 97.40 |
| 16 | 0.53 | 12.95 | 0.00 | 9.88 | 0.00 | 0.00 | 0.79 | 0.00 | 0.07 | 0.06 | 0.00 | 75.72 |
| 17 | 3.15 | 37.14 | 0.02 | 20.76 | 0.00 | 0.05 | 1.00 | 0.00 | 0.07 | 0.09 | 0.00 | 37.74 |
| 18 | 2.52 | 37.02 | 0.00 | 18.69 | 0.01 | 0.00 | 0.84 | 0.00 | 0.04 | 0.12 | 0.00 | 40.76 |
| 19 | 1.77 | 26.17 | 0.13 | 22.43 | 0.11 | 0.40 | 1.74 | 1.68 | 0.10 | 0.26 | 0.00 | 45.20 |
| 20 | 2.02 | 32.97 | 0.08 | 22.60 | 0.07 | 0.19 | 2.42 | 0.00 | 0.09 | 0.29 | 0.00 | 39.26 |
| 21 | 1.28 | 23.44 | 0.00 | 22.22 | 0.03 | 0.00 | 5.01 | 0.00 | 0.10 | 0.61 | 0.00 | 47.31 |
| 22 | 1.40 | 28.47 | 0.15 | 21.27 | 0.04 | 0.40 | 5.00 | 0.00 | 0.11 | 0.47 | 0.00 | 42.70 |
| 23 | 1.76 | 27.84 | 0.01 | 23.25 | 0.06 | 0.09 | 4.78 | 0.00 | 0.12 | 0.53 | 0.00 | 41.57 |
| 24 | 5.02 | 43.24 | 1.03 | 27.20 | 0.01 | 0.69 | 1.93 | 0.00 | 0.05 | 0.19 | 0.00 | 20.65 |
| 25 | 5.65 | 61.07 | 0.25 | 25.12 | 0.57 | 0.54 | 0.00 | 0.00 | 0.07 | 0.00 | 0.00 | 6.72 |
| 26 | 11.30 | 48.96 | 7.52 | 26.50 | 2.30 | 2.24 | 0.00 | 0.00 | 0.07 | 0.00 | 0.00 | 1.09 |
| 27 | 21.75 | 43.26 | 11.75 | 8.83 | 1.92 | 3.95 | 1.29 | 0.55 | 0.02 | 0.08 | 0.00 | 6.61 |
| 28 | 29.83 | 32.36 | 20.28 | 10.23 | 1.16 | 6.09 | 0.04 | 0.01 | 0.00 | 0.00 | 0.00 | 0.00 |
| 29 | 13.44 | 66.05 | 6.99 | 8.94 | 1.62 | 2.88 | 0.03 | 0.04 | 0.00 | 0.01 | 0.00 | 0.00 |
| 30 | 12.10 | 60.71 | 9.93 | 9.18 | 3.52 | 2.60 | 1.57 | 0.02 | 0.00 | 0.36 | 0.00 | 0.00 |
| 31 | 15.54 | 39.56 | 26.08 | 7.91 | 9.60 | 1.06 | 0.02 | 0.13 | 0.00 | 0.05 | 0.05 | 0.00 |
| 32 | 11.57 | 38.59 | 15.86 | 10.70 | 4.71 | 1.10 | 3.83 | 0.24 | 0.19 | 0.27 | 0.17 | 12.77 |
| 33 | 6.28 | 31.22 | 1.51 | 13.45 | 3.20 | 0.03 | 7.70 | 0.00 | 0.20 | 0.49 | 0.00 | 35.93 |
| 34 | 2.22 | 30.97 | 6.48 | 44.70 | 11.76 | 3.83 | 0.00 | 0.00 | 0.00 | 0.01 | 0.03 | 0.00 |
| 35 | 4.67 | 38.53 | 5.27 | 48.08 | 0.00 | 3.39 | 0.04 | 0.00 | 0.00 | 0.00 | 0.00 | 0.00 |
| 36 | 5.00 | 33.95 | 8.60 | 49.43 | 0.01 | 3.01 | 0.00 | 0.00 | 0.00 | 0.00 | 0.00 | 0.00 |
| 37 | 11.56 | 31.27 | 17.21 | 35.43 | 0.01 | 4.48 | 0.02 | 0.00 | 0.00 | 0.00 | 0.00 | 0.00 |
| 38 | 7.36 | 32.72 | 8.70 | 48.61 | 0.00 | 2.58 | 0.03 | 0.00 | 0.00 | 0.00 | 0.00 | 0.00 |
| 39 | 22.41 | 23.06 | 27.58 | 24.68 | 0.00 | 2.24 | 0.01 | 0.00 | 0.00 | 0.00 | 0.00 | 0.00 |
| 40 | 33.21 | 16.09 | 34.70 | 12.04 | 0.01 | 3.92 | 0.03 | 0.00 | 0.00 | 0.00 | 0.00 | 0.00 |
| 41 | 37.51 | 8.46 | 46.04 | 5.94 | 0.01 | 2.02 | 0.02 | 0.00 | 0.00 | 0.00 | 0.00 | 0.00 |
| 42 | 35.73 | 23.68 | 17.33 | 11.73 | 0.05 | 11.39 | 0.09 | 0.00 | 0.00 | 0.00 | 0.00 | 0.00 |
| 43 | 39.26 | 16.11 | 25.66 | 10.40 | 0.03 | 8.45 | 0.09 | 0.00 | 0.00 | 0.00 | 0.00 | 0.00 |
| 44 | 43.62 | 11.89 | 25.87 | 13.03 | 0.02 | 5.53 | 0.03 | 0.00 | 0.00 | 0.00 | 0.00 | 0.00 |
| 45 | 37.97 | 14.69 | 21.47 | 14.57 | 0.06 | 11.14 | 0.10 | 0.00 | 0.00 | 0.00 | 0.00 | 0.00 |
| 46 | 35.30 | 9.80 | 47.75 | 3.48 | 0.00 | 3.65 | 0.00 | 0.00 | 0.00 | 0.00 | 0.00 | 0.00 |
| 47 | 41.13 | 10.89 | 40.21 | 2.25 | 0.00 | 5.51 | 0.00 | 0.00 | 0.00 | 0.00 | 0.00 | 0.00 |
| 48 | 41.92 | 29.90 | 7.92 | 6.88 | 0.00 | 13.34 | 0.04 | 0.00 | 0.00 | 0.00 | 0.00 | 0.00 |
| 49 | 40.81 | 19.31 | 28.92 | 6.36 | 0.01 | 4.58 | 0.02 | 0.00 | 0.00 | 0.00 | 0.00 | 0.00 |
| 50 | 39.20 | 13.92 | 34.11 | 9.95 | 0.03 | 2.75 | 0.03 | 0.00 | 0.00 | 0.00 | 0.00 | 0.00 |
| 51 | 36.78 | 18.53 | 31.44 | 9.85 | 0.03 | 3.31 | 0.06 | 0.00 | 0.00 | 0.00 | 0.00 | 0.00 |
| 52 | 42.95 | 14.23 | 33.32 | 7.15 | 0.01 | 2.32 | 0.02 | 0.00 | 0.00 | 0.00 | 0.00 | 0.00 |
| 53 | 39.57 | 17.93 | 29.86 | 8.31 | 0.03 | 4.23 | 0.07 | 0.00 | 0.00 | 0.00 | 0.00 | 0.00 |

*S. salsa* marsh, *Suaeda salsa*-dominated marsh; *P. australis* marsh, *Phragmites australis*-dominated marsh; *S. alterniflora* marsh, *Spartina alterniflora*-dominated marsh. For habitat type definitions, see Table S2.

(b)Spatio-temporal matrix of weekly habitat use in the adult group of red-crowned cranes

| Week | *S.salsa* marsh | *P.australis* marsh | *S.alterniflora* marsh | Water surface | Aquaculture pond | Herbaceous marsh | Farmland | Saltern | Built-up area | Forest | Mud flat | Tourism area |
| --- | --- | --- | --- | --- | --- | --- | --- | --- | --- | --- | --- | --- |
| 1 | 0.25 | 3.71 | 0.00 | 4.24 | 0.76 | 0.00 | 0.38 | 0.00 | 0.09 | 0.00 | 0.00 | 90.58 |
| 2 | 0.82 | 15.26 | 0.00 | 13.41 | 0.00 | 0.00 | 0.20 | 0.00 | 0.08 | 0.00 | 0.00 | 70.24 |
| 3 | 1.83 | 25.42 | 0.00 | 13.05 | 0.00 | 0.00 | 0.00 | 0.00 | 0.05 | 0.00 | 0.00 | 59.66 |
| 4 | 0.80 | 10.04 | 0.00 | 7.34 | 0.00 | 0.00 | 0.01 | 0.00 | 0.05 | 0.00 | 0.00 | 81.76 |
| 5 | 1.20 | 29.39 | 0.00 | 17.30 | 0.00 | 0.00 | 0.00 | 0.00 | 0.08 | 0.00 | 0.00 | 52.04 |
| 6 | 0.77 | 21.88 | 0.00 | 13.24 | 0.00 | 0.00 | 0.00 | 0.00 | 0.07 | 0.00 | 0.00 | 64.04 |
| 7 | 1.40 | 31.13 | 0.00 | 18.41 | 0.02 | 0.00 | 0.55 | 0.00 | 0.18 | 0.02 | 0.00 | 48.29 |
| 8 | 1.56 | 30.03 | 0.00 | 17.21 | 0.00 | 0.00 | 0.00 | 0.00 | 0.08 | 0.00 | 0.00 | 51.13 |
| 9 | 1.20 | 18.85 | 0.00 | 11.77 | 0.00 | 0.00 | 0.01 | 0.00 | 0.09 | 0.00 | 0.00 | 68.08 |
| 10 | 2.62 | 27.31 | 0.00 | 12.86 | 0.01 | 0.00 | 0.01 | 0.00 | 0.05 | 0.00 | 0.00 | 57.15 |
| 11 | 1.18 | 17.11 | 0.00 | 12.14 | 0.00 | 0.00 | 0.00 | 0.00 | 0.09 | 0.00 | 0.00 | 69.47 |
| 12 | 1.18 | 27.54 | 0.00 | 20.16 | 0.00 | 0.00 | 0.00 | 0.00 | 0.09 | 0.00 | 0.00 | 51.02 |
| 13 | 1.04 | 26.09 | 0.00 | 18.68 | 0.00 | 0.00 | 0.00 | 0.00 | 0.10 | 0.00 | 0.00 | 54.09 |
| 14 | 0.49 | 11.77 | 0.00 | 10.00 | 0.03 | 0.00 | 0.15 | 0.00 | 0.06 | 0.00 | 0.00 | 77.49 |
| 15 | 0.00 | 0.11 | 0.00 | 2.21 | 0.00 | 0.00 | 0.00 | 0.00 | 0.00 | 0.00 | 0.00 | 97.68 |
| 16 | 2.04 | 40.35 | 0.00 | 21.14 | 0.80 | 0.60 | 3.05 | 0.00 | 0.05 | 1.63 | 0.00 | 30.34 |
| 17 | 4.32 | 82.77 | 0.00 | 9.69 | 0.00 | 0.39 | 0.24 | 0.00 | 0.00 | 0.00 | 0.00 | 2.58 |
| 18 | 1.49 | 48.82 | 0.00 | 35.44 | 6.06 | 2.63 | 0.54 | 0.00 | 0.00 | 0.03 | 0.00 | 4.99 |
| 19 | 1.04 | 48.03 | 0.00 | 36.61 | 10.40 | 3.10 | 0.66 | 0.00 | 0.06 | 0.11 | 0.00 | 0.00 |
| 20 | 0.97 | 56.52 | 0.00 | 31.18 | 6.76 | 3.18 | 1.35 | 0.00 | 0.00 | 0.04 | 0.00 | 0.00 |
| 21 | 1.27 | 51.42 | 0.00 | 35.58 | 7.37 | 3.50 | 0.82 | 0.00 | 0.00 | 0.03 | 0.00 | 0.00 |
| 22 | 1.20 | 49.99 | 0.00 | 35.43 | 8.15 | 3.82 | 1.35 | 0.00 | 0.00 | 0.06 | 0.00 | 0.00 |
| 23 | 0.85 | 37.11 | 0.00 | 38.09 | 21.86 | 1.10 | 0.65 | 0.00 | 0.00 | 0.33 | 0.00 | 0.00 |
| 24 | 0.83 | 37.76 | 0.00 | 36.06 | 23.08 | 1.26 | 0.70 | 0.00 | 0.00 | 0.32 | 0.00 | 0.00 |
| 25 | 0.83 | 37.29 | 0.00 | 37.27 | 22.34 | 1.23 | 0.69 | 0.00 | 0.00 | 0.34 | 0.00 | 0.00 |
| 26 | 0.76 | 38.67 | 0.00 | 37.05 | 21.10 | 1.50 | 0.62 | 0.00 | 0.00 | 0.28 | 0.00 | 0.00 |
| 27 | 0.83 | 43.44 | 0.00 | 38.42 | 15.24 | 1.64 | 0.15 | 0.00 | 0.00 | 0.28 | 0.00 | 0.00 |
| 28 | 0.79 | 37.28 | 0.00 | 38.26 | 21.71 | 1.15 | 0.46 | 0.00 | 0.00 | 0.33 | 0.00 | 0.00 |
| 29 | 0.86 | 45.54 | 0.00 | 38.06 | 12.66 | 2.22 | 0.44 | 0.00 | 0.00 | 0.22 | 0.00 | 0.00 |
| 30 | 0.94 | 53.63 | 0.00 | 40.12 | 0.94 | 4.38 | 0.00 | 0.00 | 0.00 | 0.00 | 0.00 | 0.00 |
| 31 | 1.31 | 52.82 | 0.00 | 41.55 | 0.59 | 3.71 | 0.00 | 0.00 | 0.00 | 0.00 | 0.00 | 0.00 |
| 32 | 1.02 | 50.86 | 0.00 | 43.13 | 1.05 | 3.94 | 0.00 | 0.00 | 0.00 | 0.00 | 0.00 | 0.00 |
| 33 | 1.02 | 50.78 | 0.00 | 43.42 | 0.89 | 3.89 | 0.00 | 0.00 | 0.00 | 0.00 | 0.00 | 0.00 |
| 34 | 1.04 | 51.64 | 0.00 | 42.55 | 0.95 | 3.81 | 0.00 | 0.00 | 0.00 | 0.00 | 0.00 | 0.00 |
| 35 | 1.43 | 54.16 | 0.00 | 40.16 | 0.50 | 3.66 | 0.08 | 0.00 | 0.00 | 0.00 | 0.00 | 0.00 |
| 36 | 1.06 | 50.54 | 0.00 | 43.83 | 0.87 | 3.70 | 0.00 | 0.00 | 0.00 | 0.00 | 0.00 | 0.00 |
| 37 | 1.07 | 50.47 | 0.00 | 43.67 | 1.18 | 3.59 | 0.01 | 0.00 | 0.00 | 0.00 | 0.00 | 0.00 |
| 38 | 1.78 | 53.42 | 0.00 | 41.57 | 0.31 | 2.92 | 0.00 | 0.00 | 0.00 | 0.00 | 0.00 | 0.00 |
| 39 | 2.22 | 54.68 | 0.00 | 40.35 | 0.12 | 2.64 | 0.00 | 0.00 | 0.00 | 0.00 | 0.00 | 0.00 |
| 40 | 1.67 | 53.50 | 0.00 | 41.21 | 0.40 | 2.96 | 0.00 | 0.00 | 0.00 | 0.00 | 0.00 | 0.25 |
| 41 | 1.96 | 49.03 | 0.00 | 43.52 | 0.75 | 4.72 | 0.01 | 0.00 | 0.00 | 0.01 | 0.00 | 0.00 |
| 42 | 2.34 | 50.92 | 0.00 | 41.12 | 0.22 | 5.40 | 0.00 | 0.00 | 0.00 | 0.00 | 0.00 | 0.00 |
| 43 | 2.38 | 54.32 | 0.00 | 37.16 | 0.16 | 5.97 | 0.00 | 0.00 | 0.00 | 0.00 | 0.00 | 0.00 |
| 44 | 4.14 | 34.00 | 0.00 | 49.58 | 0.00 | 11.86 | 0.41 | 0.00 | 0.00 | 0.02 | 0.00 | 0.00 |
| 45 | 2.87 | 49.51 | 0.00 | 40.41 | 0.07 | 7.15 | 0.00 | 0.00 | 0.00 | 0.00 | 0.00 | 0.00 |
| 46 | 3.66 | 38.44 | 0.00 | 48.02 | 0.01 | 9.86 | 0.00 | 0.00 | 0.00 | 0.00 | 0.00 | 0.00 |
| 47 | 3.30 | 38.50 | 0.00 | 47.80 | 0.01 | 10.39 | 0.00 | 0.00 | 0.00 | 0.00 | 0.00 | 0.00 |
| 48 | 3.41 | 38.59 | 0.00 | 47.49 | 0.11 | 10.41 | 0.00 | 0.00 | 0.00 | 0.00 | 0.00 | 0.00 |
| 49 | 1.96 | 55.92 | 0.00 | 36.67 | 0.37 | 5.08 | 0.00 | 0.00 | 0.00 | 0.00 | 0.00 | 0.00 |
| 50 | 1.77 | 61.21 | 0.00 | 32.41 | 0.26 | 4.34 | 0.00 | 0.00 | 0.00 | 0.00 | 0.00 | 0.00 |
| 51 | 2.22 | 57.97 | 0.00 | 34.19 | 0.12 | 5.51 | 0.00 | 0.00 | 0.00 | 0.00 | 0.00 | 0.00 |
| 52 | 3.01 | 43.82 | 0.00 | 45.77 | 0.98 | 6.42 | 0.00 | 0.00 | 0.00 | 0.00 | 0.00 | 0.00 |
| 53 | 2.61 | 47.81 | 0.00 | 45.41 | 1.04 | 3.07 | 0.00 | 0.00 | 0.00 | 0.07 | 0.00 | 0.00 |

*S. salsa* marsh, *Suaeda salsa*-dominated marsh; *P. australis* marsh, *Phragmites australis*-dominated marsh; *S. alterniflora* marsh, *Spartina alterniflora*-dominated marsh. For habitat type definitions, see Table S2.

**Construction of the weekly spatio-temporal matrix for stPCA:** Buffer zones were first constructed around the hourly GPS locations of red-crowned cranes. Within each buffer, the area proportions of 12 major land-cover types were extracted and used as proxy variables for habitat resource availability. These habitat-type proportions were then summarized at the weekly scale for each individual to characterize weekly habitat use. To improve the robustness of comparisons between age classes under a limited sample size, individuals No. 1, No. 2, and No. 6 were assigned to the immature group, whereas individuals No. 3, No. 4, and No. 5 were assigned to the adult group. For each week, the median proportion of each habitat type was calculated across all individuals within the same age group. These weekly median values were used as representative group-level habitat-use values and were subsequently used to construct the spatio-temporal matrix for stPCA.

In the resulting matrix, rows represented weeks and columns represented the 12 land-cover variables within the buffer zone (Table S3). The matrix was standardized prior to PCA, and principal component extraction was then performed to identify the dominant ecological gradients underlying habitat-use variation. Weekly aggregation was adopted to balance temporal sensitivity and analytical stability, while reducing the influence of short-term fluctuations and the spatiotemporal autocorrelation associated with high-frequency GPS observations. The use of medians further reduced the effect of short-term extreme values from individual cranes, thereby providing a more robust representation of age-specific habitat-use dynamics during the early post-release stage.
